# Functional and expression analyses of kiwifruit *SOC1*-like genes suggest that they may not have a role in the transition to flowering but may affect the duration of dormancy

**DOI:** 10.1093/jxb/erv234

**Published:** 2015-05-15

**Authors:** Charlotte Voogd, Tianchi Wang, Erika Varkonyi-Gasic

**Affiliations:** The New Zealand Institute for Plant & Food Research Limited (Plant & Food Research), Mt Albert, Private Bag 92169, Auckland 1142, New Zealand

**Keywords:** *Actinidia*, budbreak, dormancy, flowering SOC1.

## Abstract

The MADS-domain transcription factor *SUPPRESSOR OF OVEREXPRESSION OF CONSTANS1* (*SOC1*) is one of the key integrators of endogenous and environmental signals that promote flowering in the annual species *Arabidopsis thaliana*. In the deciduous woody perennial vine kiwifruit (*Actinidia* spp.), environmental signals are integrated to regulate annual cycles of growth and dormancy. Accumulation of chilling during winter is required for dormancy break and flowering in spring. In order to understand the regulation of dormancy and flowering in kiwifruit, nine kiwifruit *SOC1*-like genes were identified and characterized. All genes affected flowering time of *A. thaliana* Col-0 and were able to rescue the late flowering phenotype of the *soc1-2* mutant when ectopically expressed. A differential capacity for homodimerization was observed, but all proteins were capable of strong interactions with SHORT VEGETATIVE PHASE (SVP) MADS-domain proteins. Largely overlapping spatial domains but distinct expression profiles in buds were identified between the *SOC1*-like gene family members. Ectopic expression of *AcSOC1e*, *AcSOC1i*, and *AcSOC1f* in *Actinidia chinensis* had no impact on establishment of winter dormancy and failed to induce precocious flowering, but *AcSOC1i* reduced the duration of dormancy in the absence of winter chilling. These findings add to our understanding of the *SOC1*-like gene family and the potential diversification of *SOC1* function in woody perennials.

## Highlight

The expanded kiwifruit *SOC1*-like gene family causes early flowering in *Arabidopsis* and not kiwifruit, but may affect duration of kiwifruit dormancy, potentially by interaction with *SVP*.

## Introduction

The transition from vegetative to reproductive development in plants is regulated by both endogenous signals and environmental cues. In the annual *Arabidopsis thaliana*, *FLOWERING LOCUS T* (*FT*), *SUPPRESSOR OF OVEREXPRESSION OF CONSTANS1* (*SOC1*), and *LEAFY* (*LFY*) integrate these signals from multiple pathways to promote the transition to flowering. The *SOC1* gene has been well characterized (Borner *et al.*, 2000; Samach *et al.*, 2000; Moon *et al.*, 2003, 2005) and encodes a type II MADS-box transcription factor that is thought to exert its action by promoting expression of *LFY* through binding to its promoter (Liu *et al.*, 2008; Lee *et al.*, 2008). *SOC1* is positively regulated by *CONSTANS* (*CO*), *FT* (Hepworth *et al.*, 2002; Yoo *et al.*, 2005; Torti *et al.*, 2012), and the age-dependent (Wang *et al.*, 2009) and gibberellin pathways (Moon *et al.*, 2003), while it is negatively regulated by *FLOWERING LOCUS C* (*FLC*) and *SHORT VEGETATIVE PHASE* (*SVP*) (Searle *et al.*, 2006; Li *et al.*, 2008; Jang *et al.*, 2009; Gregis *et al.*, 2013). SOC1 interacts with MADS box proteins including floral activators AGAMOUS-LIKE 24 (AGL24), APETALA1 (AP1), and FRUITFUL (FUL), and binds directly to regulatory sequences of several flowering MADS box genes, including floral repressors *SVP*, *AGAMOUS-LIKE15* (*AGL15*), and *AGAMOUS-LIKE18* (*AGL18*) (de Folter *et al.*, 2005; Lee *et al.*, 2008; Seo *et al.*, 2009; Immink *et al.*, 2012; Tao *et al.*, 2012; Balanzà *et al.*, 2014). A complex regulatory network is thus established among *SOC1* and other key genes that determines the integration of flowering signals, prevents floral reversion, stops premature differentiation of the floral meristem, and regulates floral patterning (Liu *et al.*, 2007, 2009; Melzer *et al.*, 2008; Lee and Lee, 2010, Pose *et al.*, 2012).

*Arabidopsis* contains five other *SOC1*-like genes. *AGAMOUS-LIKE 42* (*AGL42*), *AGAMOUS-LIKE 71* (*AGL71*), *AGAMOUS-LIKE 72* (*AGL72*), and root-expressed *AGAMOUS-LIKE 19* (*AGL19*) (Alvarez-Buylla *et al.*, 2000) have all been implicated in floral transition (Schönrock *et al.*, 2006; Dorca-Fornell *et al.*, 2011; Kim *et al.*, 2013), while *AGAMOUS-LIKE 14* (*AGL14*), which is also preferentially expressed in root tissues, modulates auxin transport during root development (Garay-Arroyo *et al.*, 2013).

*SOC1*-like genes have been described in both gymnosperms (Tandre *et al.*, 1995; Winter *et al.*, 1999; Uddenberg *et al.*, 2013; Katahata *et al.*, 2014) and angiosperms (Menzel *et al.*, 1996; Cseke *et al.*, 2003; Ferrario *et al.*, 2004; Lee *et al.*, 2004; Watson and Brill, 2004; Nakamura *et al.*, 2005; Tan and Swain, 2007; Papaefthimiou *et al.*, 2012; Zhou *et al.*, 2013), but functional data are limited. Although *SOC1*-like genes have been shown to be preferentially expressed in vegetative tissues, some are expressed in reproductive organs (Decroocq *et al.*, 1999; Heuer *et al.*, 2001; Ruokolainen *et al.*, 2011). Several *SOC1*-like genes have been shown to be able to accelerate flowering when overexpressed (Ferrario *et al.*, 2004; Ma *et al.*, 2011) or to delay flowering in mutants and upon silencing (Lee *et al.*, 2004; Preston *et al.*, 2014). However, diversification of function has been observed in perennials. In the perennial herb strawberry, *SOC1* represses flowering and promotes vegetative growth (Mouhu *et al.*, 2013). In apricot, a *SOC1*-like gene is implicated in regulation of winter chilling and dormancy break (Trainin *et al.*, 2013), while in aspen, a *SOC1* homologue may have a role in wood formation (Cseke *et al.*, 2003).

In the woody perennial vine kiwifruit (*Actinidia* spp.), latent buds differentiated in the previous growing season break dormancy after accumulation of winter chilling to initiate a new cycle of vegetative growth. Flowering occurs in spring as a result of differentiation of axillary meristems within latent buds, which are believed to have acquired floral fate in the previous spring–summer season (Snelgar and Manson, 1992; Snowball, 1996; Walton *et al.*, 1997). In order to understand the molecular mechanisms of flowering in kiwifruit, a study of MADS box genes with similarity to *SOC1* was undertaken. Here, the expression and functional analysis of nine kiwifruit *SOC1*-like genes are reported and their potential roles during bud and flower development are discussed.

## Materials and methods

### Plant material

Kiwifruit plant material was collected from female cultivars ‘Hort16A’ (*Actinidia chinensis* Planch.) and ‘Hayward’ [*A. deliciosa* (A. Chev.) C.F. Liang et A.R. Ferguson]. Spatial expression analysis was performed on a set of *A. chinensis* cDNA samples described in Ledger *et al.* (2010), which included tissues from leaf, stem, bud, root, flower, young fruit, mature fruit, and seed. For temporal gene expression analysis during the annual cycle of bud and flower development, the *A. deliciosa* samples collected near Hamilton, described by Walton *et al.* (2001), and *A. deliciosa* samples collected near Kerikeri, described by Wu *et al.* (2012), were used. Daily expression analysis was performed on *A. chinensis* leaf cDNA samples described by Varkonyi-Gasic *et al.* (2013). For RNA ligase-mediated 5ʹ rapid amplification of cDNA ends (5ʹ RACE) and amplification of full-length coding sequences, breaking buds were collected from *A. chinensis* canes grown in natural field conditions at the ‘Punchbowl’ kiwifruit orchard in Pukekohe, New Zealand in August 2010. Combined buds were frozen in liquid nitrogen and stored at –80 °C until needed.

### Gene isolation and vector construction

Gene-specific oligonucleotide primers were designed based on available sequence data. The 5ʹ regions of *AcSOC1b*, *AcSOC1e*, and *AcSOC1i* were absent from the expressed sequence tag (EST) database and were isolated by 5ʹ RACE. A cDNA library was made from *A. chinensis* bud RNA using the GeneRacer™ kit (Invitrogen) according to the manufacturer’s instructions and the primers provided. Gene-specific primer sequences are presented in Supplementary Table S1 available at *JXB* online. Amplified products were cloned into *Sma*I-digested pUC19 and verified by sequence analysis. Full-length coding sequences of all kiwifruit *SOC1*-like genes were then amplified using a two-step adaptor PCR strategy which incorporated the complete *att*B1 and *att*B2 sequence at the 5ʹ and 3ʹ end, respectively (Supplementary Table S1). Purified PCR fragments were each recombined in the Gateway™ pDONR221 vector (Invitrogen), resulting in entry clones. Entry clones were verified using sequence analysis and then recombined into pHYGREX5, which placed each cDNA between the *Cauliflower mosiac virus* (CaMV) 35S promoter and the ocs 3ʹ transcriptional terminator. pHYGREX5, a Gateway-adapted version of the binary vector pCAMBIA1300 was constructed by isolating the 3.9kb 35S-*att*R1-Cm-*ccd*B-*att*R2-OCS cassette from pHEX2 (Hellens *et al.*, 2005) by *Sac*I/*Sac*II digestion, gel purification, and ligation into *Sma*I-digested pCAMBIA1300. The orientation of the cassette is such that the 35S-Gateway-OCS cassette is in the opposite orientation from the 35S-HPTII unit. The resulting plasmids were transformed into *Agrobacterium tumefaciens* strain GV3101 by electroporation. *AcSOC1e*, *AcSOC1i*, and *AcSOC1f* entry clones were also recombined into the 35S-Gateway-OCS cassette of pHEX2 (Hellens *et al.*, 2005) and transformed into *A. tumefaciens* strain EHA105 by electroporation.

### Identification and phylogenetic study of kiwifruit *SOC1*-like genes

*Actinidia* cDNA sequences with homology to the *Arabidopsis SOC1* clade (*SOC1*, *AGL14*, *AGL19*, *AGL42*, *AGL71*, and *AGL72*; TAIR, http://www.arabidopsis.org/) were identified in the Plant & Food Research EST database (Crowhurst *et al.*, 2008) using BLAST alignment (Altschul *et al.*, 1997). Sequences from species other than kiwifruit and *Arabidopsis* were obtained from the GenBank DNA database (http://www.ncbi.nlm.nih.gov/genbank/).

Following sequence alignment of the full-length deduced amino acid sequences, a phylogenetic tree was calculated with the Geneious Tree Builder plug-in from Geneious 5.5.6 (Drummond *et al.*, 2011) using the Neighbor–Joining method and the bootstrap confidence test (1000 replicates). *Arabidopsis* AGL6 was included as an outgroup.

### RNA extractions and cDNA synthesis

Total RNA was extracted from frozen kiwifruit bud tissue as described by Stiekema *et al.* (1988), except that the RNA extraction buffer contained 50mM TRIS pH 8.0, 10mM EDTA, 0.4M LiCl, 1% SDS, and 0.8% β-mercaptoethanol. In addition, the phenol used was buffered to pH 7.0, the chloroform contained indole acetic acid (IAA; 24:1), and the final LiCl concentration for precipitation and washing was 2.5M. Total RNA was extracted from transgenic kiwifruit leaves according to the method of Chang *et al.* (1993). Total RNA from wild-type and transgenic *Arabidopsis* leaves was isolated using Trizol reagent (Invitrogen) according to the manufacturer’s instructions. A 1 μg aliquot of total RNA was treated with DNase I, then reverse-transcribed using Superscript III (Invitrogen) according to the manufacturer’s instructions.

### Quantitative real-time PCR (qRT-PCR) analysis

Gene-specific primers for qRT-PCR of endogenous kiwifruit genes were designed to include preferably a portion of the 3ʹ-untranslated region (UTR), and to amplify products between 96bp and 118bp in size; primers for qRT-PCR of kiwifruit transgene coding regions were designed to amplify products between 97bp and 140bp (Supplementary Table S1 at *JXB* online). RT-PCRs were performed using FastStart DNA Master^PLUS^ SYBR Green I reaction mix on a LightCycler^®^ 1.5 instrument (Roche). A non-template control was included in each run. Amplification was carried out with an initial denaturing step at 95 °C for 5min, then 40–50 cycles of 95 °C for 5 s, 60 °C for 5 s, and 72 °C for 10 s. The PCR efficiency for each individual sample was calculated using the LinRegPCR 11.1 software (Ramakers *et al.*, 2003; Ruijter *et al.*, 2009). The mean efficiency per amplicon was then included in the calculation of relative expression ratios according to the comparative cycle threshold method (Pfaffl, 2001). Ct values were determined using the second derivative maximum method in the LightCycler^®^ 480 software 1.5.0. For any given set of kiwifruit samples, expression of the commonly used reference genes *GAPDH*, *ACT*, *EF1α*, *UBC9*, and *PP2A* (Crowhurst *et al.*, 2008) was analysed using GeNORM software (Vandesompele *et al.*, 2002) to identify the most stably expressed gene. *ACT2* (At3g18780) was used as a reference gene for transgenic *Arabidopsis* analyses. For primer sequences of reference genes, see Supplementary Table S1. Representative PCR products for each amplicon were verified by sequence analysis.

### Generation of transgenic *Arabidopsis* plants

Overexpression of kiwifruit genes in *Arabidopsis* was carried out in wild-type ecotype Col-0 and in the Col-0 *soc1-2* mutant (Lee *et al.*, 2000) using pHYGREX5-based constructs. Genomic DNA was isolated according to Edwards *et al.* (1991), genotyping of the wild-type and mutant *soc1-2* allele was performed with PCR primers based on those of Moon *et al.* (2005), and the presence of the transgene was confirmed by PCR using oligonucleotide primer RPH144, specific to the 35S promoter, and HYG-rev, specific to the hygromycin phosphotransferase gene. Oligonucleotide primer sequences used for genotyping are presented in Supplementary Table S1 at *JXB* online. *Agrobacterium tumefaciens*-mediated plant transformation was performed by the floral dipping method (Clough and Bent, 1998). Transformed seedlings were selected on half-strength Murashige and Skoog (MS) agar containing 25 mgl^–1^ hygromycin, then transferred to soil. Plants were grown in a controlled environment room at 20 °C under non-inductive short-day conditions (8:16h, light:dark). Flowering time was recorded and expressed as the number of rosette leaves when the primary inflorescence was 0.5cm long.

### Generation and growth of transgenic *Actinidia* plants

Three kiwifruit *SOC1*-like genes, *AcSOC1e*, *AcSOC1i*, and *AcSOC1f*, were each transformed into *A. chinensis* using pHEX2-based constructs. The transformation procedures were as previously described (Wang *et al.*, 2007). Calli which formed in the regeneration and selection medium containing 150 mgl^–1^ kanamycin were excised individually and transferred to fresh regeneration and selection medium for bud induction. More than 10 independent transgenic lines were produced for each of the three *SOC1*-like genes. After rooting, these transgenic plants were potted and grown in ambient conditions in the containment glasshouse at Plant & Food Research, Mt Albert, Auckland, New Zealand for 11 months. In the winter, plants were sprayed with 6% (w/v) copper sulphate solution on 20 June 2014 to induce leaf drop. On 26 June 2014, canes were excised and immediately dissected into single-node cuttings or chilled at 4 °C for 4 weeks before dissecting into single-node cuttings. This method has been devised by Snowball (1997) to reduce variability in budbreak on whole plants caused by interaction between shoot buds on the cane (Wu *et al.*, 2014), and was used before to compare chilling requirement of *Actinidia* species (Varkonyi-Gasic *et al.*, 2013). Three single-node cuttings from chilled and unchilled cane were used per plant. The lower ends of cuttings were immersed in water and maintained at 20 °C, 16h light, 8h dark, 70–80% humidity. The number of days until visible budbreak was recorded, and the cuttings were monitored for another 4 weeks during shoot outgrowth for flowering.

### Yeast two-hybrid assay

cDNAs of the nine kiwifruit *SOC1*-like genes were recombined from entry clones into the GATEWAY destination vectors pDEST32 (pBDGAL4, bait) and pDEST22 (pADGAL4, prey) (Invitrogen). These vectors, in addition to similar vectors expressing *Arabidopsis SOC1*, *AGL24*, and *SVP* and kiwifruit *SVP*-like genes (Wu *et al.*, 2012), were individually introduced into *Saccharomyces cerevisiae* strains PJ69-4α (bait) and PJ69-4a (prey) (James *et al.*, 1996) for selection on minimal media plates (WO) lacking Leu (bait) or Trp (prey), followed by mating on YPAD plates and double sequential selection on WO media lacking both Leu and Trp. Final screening was performed on media lacking Trp, Leu, and His, and supplemented with 0, 1, 3, and 5mM 3-amino-1,2,4-triazole. Plates were incubated for 4 d at 20 °C and scored for growth. Reciprocal tests were performed in duplicate for all combinations.

## Results

### Identification of *Actinidia chinensis SOC1*-like genes

**Fig. 1.**
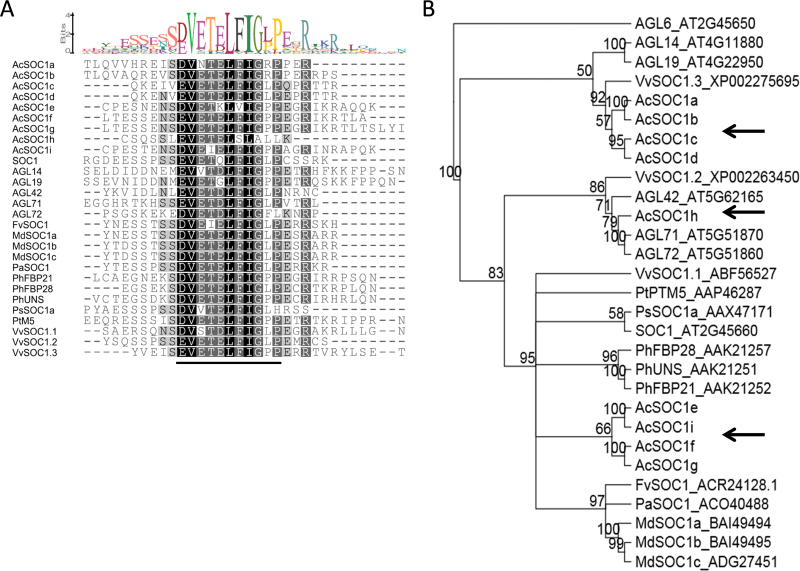
Phylogenetic analysis of kiwifruit SOC1-like protein sequences. (A) Alignment of the C-terminal region of SOC1-like amino acid sequences. The amino acid residues in the alignment are shaded according to their similarity scores: white on black, 100% similar; white on grey, 80–100% similar; black on grey, 60–80% similar; grey on white, <60% similar. The conserved SOC1 motif is underlined. The observed deviation of this motif in AcSOC1h appears to be due to a putative 1bp deletion within the motif leading to a frameshift from EVETELFFGLA to EVETELSLALL. (B) Phylogenetic tree based on the amino acid alignment of kiwifruit SOC1-like predicted proteins and SOC1-like proteins from other plant species. The tree was generated using the Geneious Tree Builder plug-in (Geneious 5.5.6) using the Neighbor–Joining method with *Arabidopsis* AGL6 as an outgroup. Numbers below the branches represent bootstrap support values from 1000 replicates (values ≥50% are indicated). Origins of the genes, with the exception of *Arabidopsis*, are indicated by a prefix as follows: *Ac*, *Actinidia chinensis*; *Vv*, *Vitis vinifera*; *Pt*, *Populus tremuloides*; *Ps*, *Pisum sativum*; *Ph*, *Petunia hybrida*; *Fv*, *Fragaria vesca*; *Pa*, *Prunus armeniaca*; *Md*, *Malus×domestica*; their GenBank accession numbers are indicated to the right. *Arabidopsis* genes *SOC1*, *AGL14*, *AGL19*, *AGL42*, *AGL71*, and *AGL72* are associated with their unique IDs. Kiwifruit *SOC1*-like genes in different clades are indicated by arrows. (This figure is available in colour at *JXB* online.)

Nine transcripts with sequence homology to *Arabidopsis SOC1* were identified in the Plant & Food Research EST database (Crowhurst *et al.*, 2008), and designated *AcSOC1a* through *AcSOC1i*. 5ʹ RACE was used where necessary before PCR amplification and cloning of the complete coding sequence from *A. chinensis* bud tissue (GenBank accession nos KP407147–KP407155). Analysis of the deduced amino acid sequence revealed that the open reading frames encode a predicted protein of between 200 and 220 amino acids and each included the conserved MADS-box, I-region, K-box, and C-terminal SOC1 motif (Fig. 1A; Supplementary Fig. S1 at *JXB* online). Each coding sequence mapped to a different pseudo-chromosome (Huang *et al.*, 2013) and is encoded by seven exons. With the exception of AcSOC1h, all proteins are associated in pairs, with members sharing >80% identity. AcSOC1e, AcSOC1i, AcSOC1f, and AcSOC1g are most similar to *Arabidopsis* SOC1, sharing between 64% and 66% identity (Supplementary Table S2). Phylogenetic analysis using the full-length deduced amino acid sequences confirmed that of the nine proteins, AcSOC1e, AcSOC1i, AcSOC1f, and AcSOC1g are most closely related to SOC1. AcSOC1a, AcSOC1b, AcSOC1c, and AcSOC1d are more closely related to AGL14 and AGL19, and AcSOC1h is most closely related to AGL42, AGL71, and AGL72 (Fig. 1B). Analysis of flanking gene models in the draft genome of *A. chinensis* (Huang *et al.*, 2013) revealed homology to genes on *Arabidopsis* chromosome numbers 2, 4, and 5 in close proximity to *Arabidopsis SOC1*-like genes and confirmed the above groupings (Supplementary Table S3).

### Functional analysis in *Arabidopsis*

**Fig. 2.**
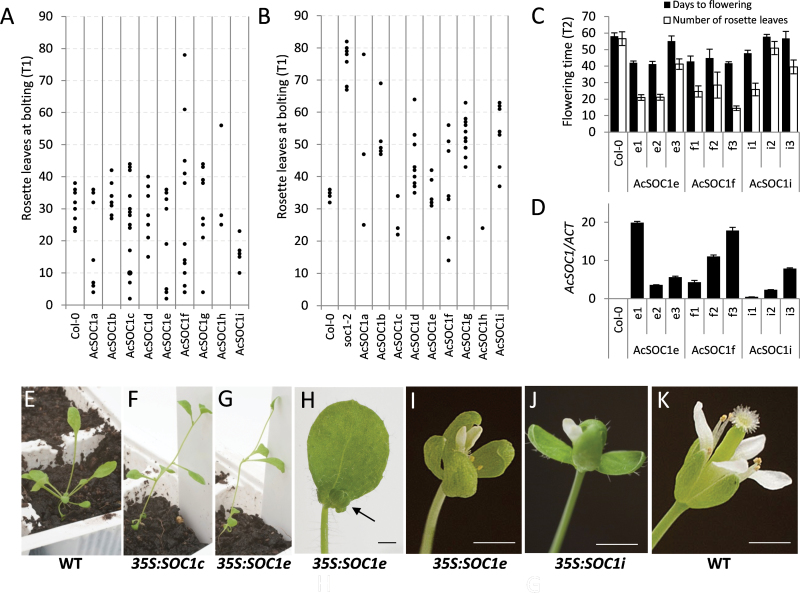
Constitutive expression of kiwifruit *SOC1*-like genes affects flowering in *Arabidopsis*. (A) Flowering time of primary transgenic (T1) *Arabidopsis* Col-0 plants grown in non-inductive short-day conditions. Flowering time was recorded as the number of rosette leaves when the primary inflorescence stems were 0.5cm long. Each dot represents one line. (B) Flowering time of T1 *Arabidopsis soc1-2* plants grown in short-day conditions, recorded and presented as above. (C) Flowering time of hygromycin-resistant progeny (T2) of three independent T1 lines of transgenic *Arabidopsis* Col-0 plants grown in short-day conditions. (D) Transgene expression in T2 plants. (E) Normal rosette development of wild-type *Arabidopsis* Col-0. (F, G) Early bolting and small rosette leaves resulting from constitutive expression of *AcSOC1* constructs. (H) Small first flower (arrow) in the *AcSOC1e* early flowering line (I, J) Abnormal flower development in lines expressing *AcSOC1* genes. (H) Wild-type *Arabidopsis* Col-0 flower. Scale bars=1mm.

To examine whether the kiwifruit *SOC1*-like genes encode functional homologues of *Arabidopsis SOC1*, their coding sequences were introduced individually into the *Arabidopsis* wild-type ecotype Col-0 and late flowering *soc1-2* mutant. Constitutive expression of all kiwifruit *SOC1*-like genes, except *AcSOC1b*, in wild-type Col-0 plants resulted in varying degrees of altered flowering time compared with that of Col-0 (Fig. 2A). All genes also showed the ability to complement the late flowering phenotype of the *soc1-2* mutant when ectopically expressed (Fig. 2B). A minimum of six hygromycin-resistant progeny of three *AcSOC1e*, *AcSOCf*, and *AcSOC1i* lines were further evaluated to confirm expression of the transgene and inheritance of the early flowering trait (Fig. 2C, D). Therefore, it is concluded that kiwifruit *SOC1*-like genes can act as floral activators in *Arabidopsis*. Early flowering plants had small rosettes (Fig. 2E–G) and often displayed altered floral development, including small flowers and flowers with large sepals and narrow sepaloid petals and carpel defects (Fig. 2H–K). Therefore, it is concluded that kiwifruit *SOC1*-like genes also impact on floral patterning.

### Expression of kiwifruit *SOC1*-like genes

**Fig. 3.**
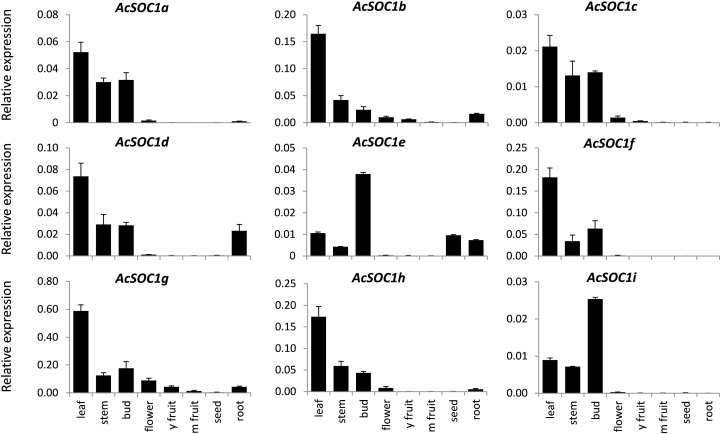
Relative expression of kiwifruit *SOC1*-like genes in leaf, stem, bud, flower, young fruit, mature fruit, seed, and root, normalized to kiwifruit *ACTIN* (*ACT*). Error bars represent standard errors (SE) for three replicates.

**Fig. 4.**
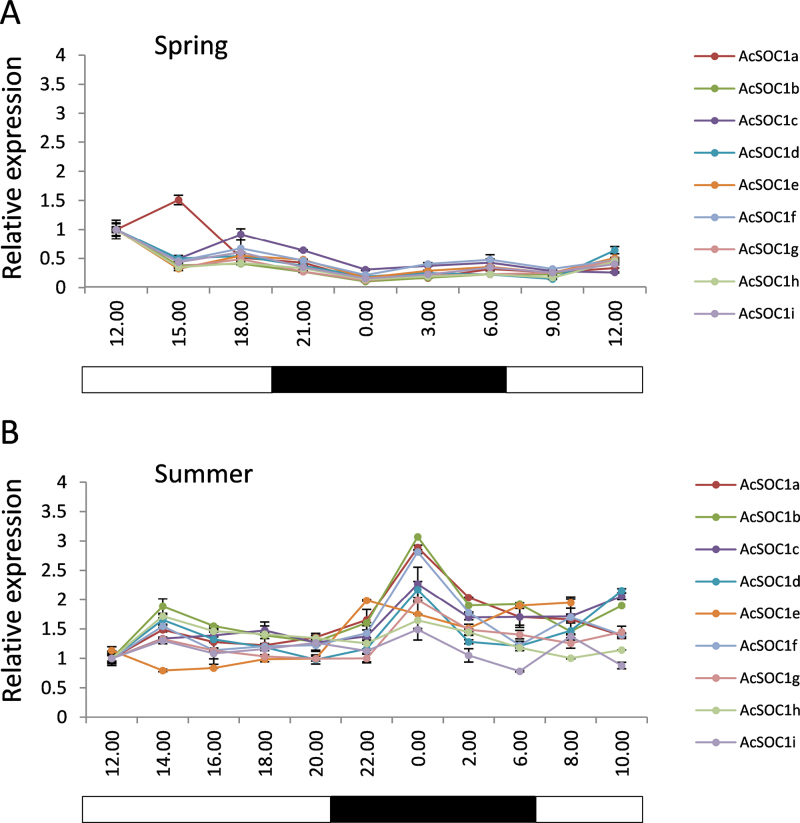
Relative expression of kiwifruit *SOC1*-like genes in leaf samples collected at regular intervals over a 24h period in spring (A) and summer (B), normalized to kiwifruit *ACTIN* (*ACT*) and expressed as a ratio to the first sample point, which was arbitrarily set to 1. Data points represent the mean ±SE for three replicates. The white and black rectangles indicate the daylight and night-time, respectively.

To associate further the biological function of the identified kiwifruit *SOC1*-like genes with specific developmental processes, their expression in eight representative vegetative and reproductive tissues of the kiwifruit plant was analysed using reverse transcription–quantitative PCR (Fig. 3). Overall, kiwifruit *SOC1*-like genes were found to have the highest expression in vegetative tissues, with *AcSOC1e* and *AcSOC1i* predominantly expressed in buds, and the remaining genes predominantly expressed in leaves. None of the *AcSOC1*-like transcripts was detected in significant quantities in fruit, and only *AcSOC1e* was detected in seeds. These findings were consistent with ESTs originating mainly from *Actinidia* leaf and bud tissues. All *AcSOC1*-like genes showed relatively stable, non-oscillating expression patterns in the leaf during the day:night cycle (Fig. 4).

**Fig. 5.**
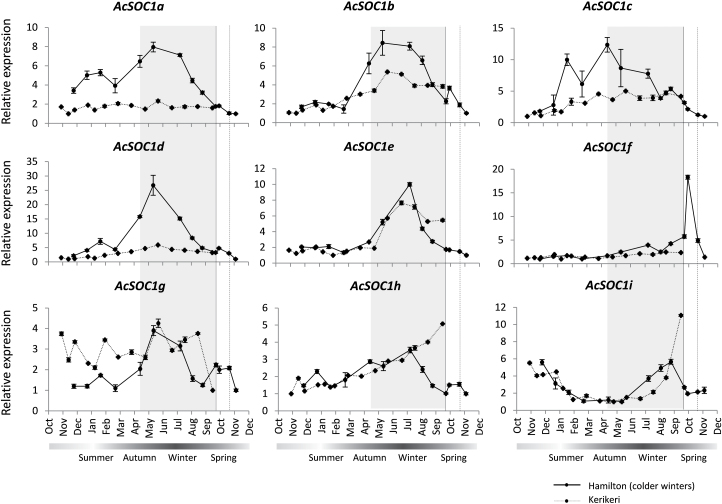
Relative expression of kiwifruit *SOC1*-like genes in axillary buds during the growth and dormancy cycles, normalized to kiwifruit *UBC9* and expressed as a ratio to the sample point with lowest expression, which was arbitrarily set to 1. Data points represent the mean ±SE for two replicates, the solid line represents samples collected from Hamilton, New Zealand, in 1995–1996, and the dotted line represents samples collected from Kerikeri, New Zealand, in 2008–2009. The vertical solid and dashed lines indicate budbreak time in Hamilton and Kerikeri, respectively. Hamilton is the region with colder winters (Supplementary Fig. S2 at *JXB* online).

Next, seasonal expression analysis was performed on lateral buds collected in regular intervals over the period of 1 year. Important developmental events, including floral evocation and bud dormancy, occur in kiwifruit buds over this period (Walton *et al.*, 2001). The sampling was performed in two different years at different locations. Distinct expression profiles were obtained for the nine kiwifruit *SOC1*-like genes (Fig. 5). *AcSOC1a*, *AcSOC1b*, *AcSOC1c*, and *AcSOC1d* genes demonstrated increased expression throughout the winter dormancy period, particularly in samples collected from the region with colder winters (Supplementary Fig. S2 at *JXB* online). A similar, yet less strong pattern was found for *AcSOC1e*, *AcSOC1g*, and *AcSOC1h*, with *AcSOC1e* peaking later in winter and *AcSOC1h* increasing after winter in one location. *AcSOC1i* expression declined during early summer of the first season, remained low during early winter, then increased and peaked late in the winter, before budbreak. The level of transcripts of *AcSOC1f* slowly but consistently rose during the winter, and a sharp peak was detected after budbreak in one location.

### Kiwifruit SOC1-like proteins interact with SVP-like proteins

**Fig. 6.**
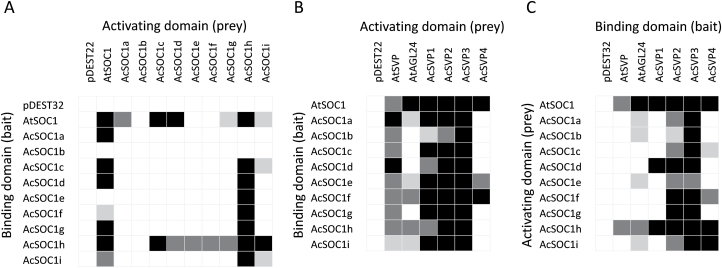
Summary of kiwifruit SOC1-like protein interactions detected by yeast two-hybrid analysis. (A) Homo- and heterodimerization of kiwifruit SOC1-like and *Arabidopsis* SOC1 proteins. (B, C) Heterodimerization of kiwifruit SOC1-like, SVP-like, and *Arabidopsis* SOC1, SVP, and AGL24. Black, very strong interaction; dark grey, strong interaction; light grey, weak interaction; white, no interaction. The summary is based on results presented in Supplementary Fig. S3 at *JXB* online.

As an important floral integrator, *Arabidopsis* SOC1 has multiple interaction partners (Pelaz *et al.*, 2001; de Folter *et al.*, 2005). It was hypothesized that the kiwifruit SOC1-like proteins also exert their function through formation of homo- and heterodimers or higher order MADS box protein complexes. In particular, heterodimerization of SOC1 and AGL24 is essential for function (Lee *et al.*, 2008), and the kiwifruit homologues of *AGL24*/ *SVP* genes are co-expressed with *SOC1*-like genes in vegetative plant tissues and show similar profiles of elevated expression over the bud dormancy period (Wu *et al.*, 2012). A yeast two-hybrid screen was therefore performed to evaluate the interaction of the kiwifruit SOC1-like proteins with each other and with kiwifruit SVP proteins. *Arabidopsis* SOC1, AGL24, and SVP were included as controls. Only *Arabidopsis* SOC1, AcSOC1h, and, to a lesser extent, AcSOC1i were capable of homodimerization in this assay. AcSOC1h strongly interacted with the majority of other AcSOC1-like proteins, which were also mostly capable of interacting with SOC1; but other interactions were not detected (Fig. 6A; Supplementary Fig. S3 at *JXB* online). On the other hand, strong or very strong interactions were observed with SVP proteins, particularly kiwifruit SVP2 and SVP3 (Fig. 6B, C; Supplementary Fig. S3).

### Ectopic expression in kiwifruit does not promote flowering but affects the duration of dormancy

**Fig. 7.**
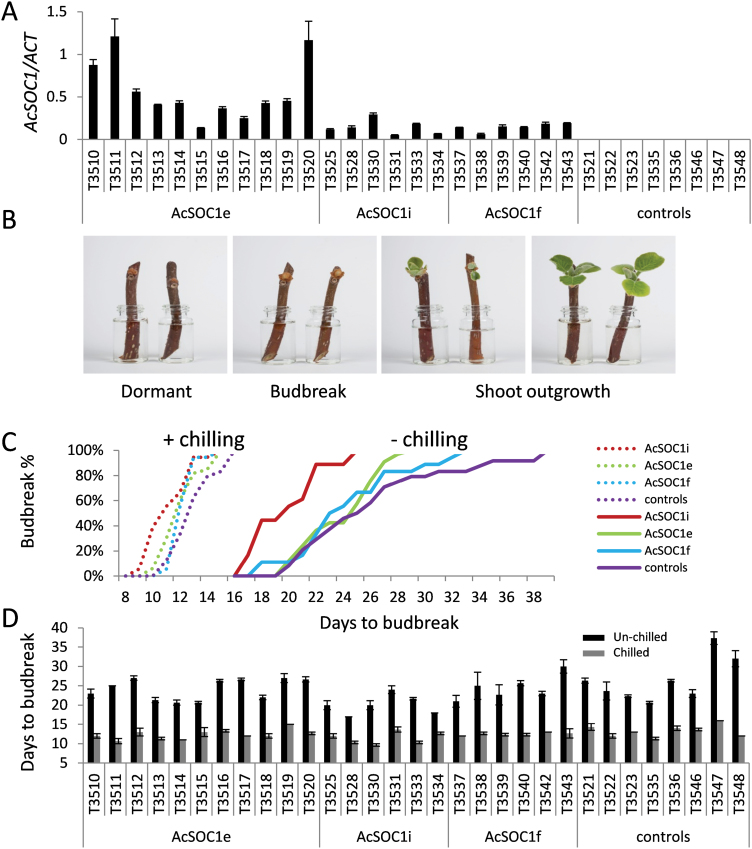
Constitutive expression of kiwifruit *SOC1*-like genes in transgenic kiwifruit *Actinidia chinensis.* (A) Relative expression of kiwifruit *SOC1*-like transgenes in *A. chinensis* transgenic lines. (B) Examples of *A. chinensis* single-node cuttings at the indicated stages. (C) Percentage of cuttings at the budbreak stage. (D) Average budbreak time of individual transgenic lines. Error bars represent SEs for three cuttings.

To investigate the role of kiwifruit *SOC1*-like genes in kiwifruit, transgenic *A. chinensis* lines were generated using the *AcSOC1e*, *AcSOC1f*, and *AcSOC1i* coding sequence driven by the CaMV *35S* promoter. These genes were chosen because of their similarity to *Arabidopsis SOC1*, high relative expression in buds (Fig. 3), and apparent sequential increase in transcription from late dormancy for *AcSOC1e*, pre-budbreak for *AcSOC1i*, and post-budbreak for *AcSOC1f* (Fig. 5). In addition, it was of interest to determine if kiwifruit *SOC1*-like genes affect the timing of the first flowering and reduce the juvenile stage. Most *Actinidia* species need several years to reach maturity and produce flowers and fruit. A minimum of eight independent lines were generated for each construct, with low to moderate transgene expression confirmed for 11 *AcSOC1e* lines and six of each of *AcSOC1i* and *AcSOC1f* lines (Fig. 7A; Supplementary Fig. S4 at *JXB* online). Normal regeneration, callus formation, and plantlet growth in tissue culture and in the soil were observed. The plants grown in the glasshouse in ambient conditions had the same appearance as controls and underwent normal cessation of active growth and leaf fall in autumn. Therefore, it is concluded that ectopic expression of *AcSOC1e*, *AcSOC1i*, and *AcSOC1f* has no impact on establishment of winter dormancy.

To investigate the effect of *SOC1*-like genes on the duration of winter dormancy and promotion of flowering, single-node cuttings from each line were collected and monitored for budbreak and flowering in controlled conditions, with and without chilling (Fig. 7B). By this method, variation in recorded budbreak is minimized, as it is less influenced by interaction between shoot buds along a cane (Snowball, 1997). In cuttings taken from control lines, budbreak occurred 20–39 d from the day the cuttings were taken, but these numbers were reduced to 17–25, 20–29, and 18–33 d in cuttings taken from *AcSOC1i*, *AcSOC1e*, and *AcSOC1f* lines, respectively (Fig. 7C). Three single-node cuttings per line were also excised from canes exposed to 4 weeks of winter chilling and further monitored for budbreak. In cuttings taken from control lines, chilling reduced the number of days to budbreak to between 11 and 15. A similar number of days was recorded for cuttings taken from lines expressing *AcSOC1* transcripts: budbreak occurred 9–15, 10–15, and 11–15 d after the cuttings were taken from chilled canes of *AcSOC1i*, *AcSOC1e*, and *AcSOC1f* lines, respectively (Fig. 7C). Analysis of the average budbreak time of cuttings from individual lines suggested that the *AcSOC1i* lines T3528 and T3534 contributed mostly to earlier budbreak time, but large variation was observed between control lines (Fig. 7D).

Neither the control nor *SOC1* transgenic plants produced flowers in the glasshouse conditions after >1 year of plant growth. Similarly, none of the cuttings from chilled or non-chilled canes flowered. Therefore, none of the three kiwifruit *SOC1*-like genes is sufficient to promote *A. chinensis* plant maturity.

## Discussion

### Expanded kiwifruit *SOC1*-like gene family

The kiwifruit genome harbours nine expressed *SOC1*-like genes. The amino acid sequences of all kiwifruit SOC1-like proteins are highly similar to those of other SOC1-like proteins and contain the consensus sequence of the SOC1 protein motif at the C-terminal end (Vandenbussche *et al.*, 2003; Nakamura *et al.*, 2005), with the exception of AcSOC1h, where the motif is disrupted as a result of a frameshift. The SOC1 motif has been suggested to play a key role in determining partner specificity in higher order complex formation (Vandenbussche *et al.*, 2003), and this mutation may be responsible for the increased homo- and heterodimerization capacity of AcSOC1h.

Phylogenetic and syntenic analyses identified that four of the kiwifruit *SOC1*-like genes (*AcSOC1a*–*AcSOC1d*) are likely orthologues of *AGL14*/*AGL19*, four (*AcSOC1e*, *AcSOC1f*, *AcSOC1g*, and *AcSOC1i*) are likely orthologues of *SOC1*, and *AcSOC1h* is most closely related to *AGL42* and *AGL71*/*AGL72*. In particular, the *AcSOC1*e and *AcSOC1i* genes are located in close proximity to an *AGL6* homologue, as they are in *Arabidopsis* and peach (Trainin *et al.*, 2013). Identification of kiwifruit representatives in each of these three *SOC1*-like subclades and association in pairs for all except *AcSOC1h* reflects the ancient triplication shared by core eudicots and two recent whole-genome duplication events in kiwifruit, which occurred after the divergence of kiwifruit from tomato and potato (Huang *et al.*, 2013) and resulted in additional gene family members. Gene loss following the two recent whole-genome duplication events is the likely reason for the presence of a single gene, *AcSOC1h*, in the *AGL42* and *AGL71*/*AGL72* subclade.

### Conservation and diversification of kiwifruit *SOC1*-like gene function

Promoted flowering observed upon overexpression of kiwifruit *SOC1*-like genes in *Arabidopsis* suggested functional conservation and a role in regulation of flowering time, but differential expression between the gene family members was indicative of functional divergence. It is possible that these genes evolved to perform similar, yet specialized functions. Similarly, although most kiwifruit *SOC1*-like protein interactions were shared between paralogues, suggestive of functional overlap, some unique interactions were also seen.

The commonality observed for all kiwifruit *SOC1*-like genes is predominant vegetative expression, a feature shared with most other *SOC1*-like genes, indicative of a common role during vegetative development as general regulators of plant organogenesis (Lee and Lee, 2010). Another commonality is the observed disturbance of normal flower development and impact on floral organ identity, probably a result of a dominant-negative interference with other factors necessary for proper floral development (Borner *et al.*, 2000; Ferrario *et al.*, 2004; Tan and Swain, 2007; Ruokolainen *et al.*, 2011). Finally, kiwifruit *SOC1*-like genes could substitute for the lack of endogenous *SOC1* when ubiquitously expressed under a strong promoter, confirming functional conservation. They were also capable of promoting flowering when expressed in wild-type *Arabidopsis*, as reported for *SOC1*-like genes from a range of plant species, including woody perennials (Sreekantan and Thomas, 2006; Tan and Swain, 2007). Curiously, some transgenic lines demonstrated delayed flowering, perhaps as a result of interference with other MADS box proteins and stochastic establishment of higher order complexes acting as floral repressors. Similarly, the levels of transgenic expression and extent of floral promotion were not always correlated.

Ectopic expression in *A. chinensis* did not result in precocity. None of the three *SOC1*-like genes was sufficient to promote flowering after the first year. Monitoring over the course of several years will reveal if any differences exist in seasonal flowering time or floral morphology. Functional characterization of *AcSOC1i* in *A. eriantha* has also been initiated. This kiwifruit species reaches maturity faster and flowers profusely in glasshouse conditions (Wang *et al.*, 2006), so the impact of this gene on flowering time, yield, and floral morphology can be evaluated in another *Actinidia* species. The same approach will be taken to establish the role of other kiwifruit *SOC1*-like genes.

### Potential role in perennial growth habit and dormancy release

Ectopic expression of *AcSOC1i*, and to a lesser extent *AcSOC1e*, combined with their expression before budbreak, suggested that these genes may have a role in regulation of the duration of dormancy in kiwifruit, acting in promotion of events leading to budbreak. On the other hand, elevated expression during earlier stages of bud dormancy for *AcSOC1a*, *AcSOC1b*, *AcSOC1c*, *AcSOC1d*, *AcSOC1g*, and *AcSOC1h* could be indicative of a role during endodormancy, perhaps reflecting metabolic and developmental processes believed to be going on in the buds during the period of winter rest (Perry, 1971).

*SOC1*-like genes have already been associated with dormancy and perennial growth habits. *SOC1* controls the growth habit in *Arabidopsis* (Melzer *et al.*, 2008), yearly cycles of vegetative and reproductive growth in strawberry (Mouhu *et al.*, 2013), and, in aspen, the *SOC1* homologue *PTM5* is implicated in seasonality and spring wood formation (Cseke *et al.*, 2003). In apricot (*Prunus armeniaca*), a *SOC1*-like gene has been associated with cold response and chilling requirements during dormancy (Olukolu *et al.*, 2009), and the gene was expressed in apricot leaves in a diurnal manner (Trainin *et al.*, 2013), perhaps reflecting the capacity to integrate temperature and photoperiodic signals required for dormancy release. In contrast, kiwifruit *SOC1*-like genes showed little response to the day:night cycle, probably because photoperiod may not have a major role in kiwifruit dormancy release and flowering (Snelgar *et al.*, 2007), although it appears to be important for dormancy establishment (Lionakis and Schwabe, 1984).

The genetic and expression studies performed in *Prunus* species were instrumental in revealing the role of another MADS box gene clade, the *SVP*-like genes, in regulation of dormancy (Bielenberg *et al.*, 2008; Yamane *et al.*, 2008, 2011; Li *et al.*, 2009; Sasaki *et al.*, 2011). A possibility exists that kiwifruit *SOC1*-like genes exert their function through interaction with *SVP* genes, in a manner similar to reports for *Arabidopsis* (Lee *et al.*, 2008; Li *et al.*, 2008; Immink *et al.*, 2012; Tao *et al.*, 2012; Gregis *et al.*, 2013). The largely overlapping spatial distribution combined with co-expression during bud dormancy (Wu *et al.*, 2012) indicate that they may be regulating each other’s transcription, while protein associations may be contributing to regulate accurate timing of kiwifruit development. Interaction with SVP3, the only kiwifruit *SVP* which does not show elevated expression during bud dormancy (Wu *et al.*, 2012) and is affecting flower development instead (Wu *et al.*, 2014), may suggest that *SOC1* genes also impact on flower development in kiwifruit.

A possible mechanism of *SOC1* action could involve control of cell division, expansion, and differentiation required for both shoot outgrowth and flowering. It is becoming clear that MADS box genes regulate processes required for all stages of organ development, including early patterning, subsequent growth, and final cellular differentiation, instead of functioning as master regulators in hierarchical networks (reviewed in Sablowski, 2015). Complex regulatory interactions enable activation of separate genetic pathways, and a recently demonstrated link with homologues of *TERMINAL FLOWER1* and gibberellin biosynthesis explain promotion of vegetative growth in strawberry (Mouhu *et al.*, 2013). A detailed analysis of kiwifruit *SOC1*-like and *SVP* pathways will therefore help to better understand woody perennial growth, and provide tools for breeding kiwifruit cultivars with different durations of dormancy.

## Supplementary data

Supplementary data are available at *JXB* online.

Figure S1. Alignment of the SOC1-like amino acid sequences.

Figure S2. Mean daily temperatures recorded during the collection of kiwifruit axillary bud samples.

Figure S3. Protein interactions detected by yeast two-hybrid analysis.

Figure S4. qRT-PCR analysis of transgenic lines using primers specific to the *AcSOC1e* and *AcSOC1i* coding sequences and primers detecting expression of the transgene.

Table S1. Oligonucleotide primer sequences used in this study.

Table S2. Percentage amino acid identity between *Arabidopsis* and kiwifruit *SOC1*-like sequences.

Table S3. Blast analysis of gene models in proximity to kiwifruit *SOC1*-like genes.

Supplementary Data
