# Supplementary material for: Functional and expression analyses of kiwifruit SOC1-like genes suggest that they may not have a role in the transition to flowering but may affect the duration of dormancy
Source: J Exp Bot. 2015 May 15;66(15):4699–710. doi: 10.1093/jxb/erv234 (PMC4507769; doi:10.1093/jxb/erv234)
Supplement: Supplementary Data [file supp_66_15_4699__index.html]

Functional and expression analyses of kiwifruit SOC1-like genes suggest that they may not have a role in the transition to flowering but may affect the duration of dormancy — Functional and expression analyses of kiwifruit SOC1-like genes suggest that they may not have a role in the transition to flowering but may affect the duration of dormancy — Supplementary Data 

# Functional and expression analyses of kiwifruit *SOC1*-like genes suggest that they may not have a role in the transition to flowering but may affect the duration of dormancy

## Supplementary Data

Data files

**Files in this Data Supplement:**

- Supplementary Data - Supplementary Data
- Supplementary Data - Supplementary Data
