# Supplementary material for: Functional and expression analyses of kiwifruit SOC1-like genes suggest that they may not have a role in the transition to flowering but may affect the duration of dormancy
Source: J Exp Bot. 2015 May 15;66(15):4699–710. doi: 10.1093/jxb/erv234 (PMC4507769; doi:10.1093/jxb/erv234)
Supplement: Supplementary Data [file supp_erv234_jexbot145029_file001.pdf]

SUPPLEMENTARY DATA

Supplementary Table S1. Oligonucleotide primer sequences used in this study.

Supplementary Table S2. Percentage amino acid identity between Arabidopsis and kiwifruit *SOC1*-like sequences.

Supplementary Table S3. Blast analysis of gene models in proximity to kiwifruit *SOC1*-like genes. Gene models upstream and downstream of the kiwifruit *SOC1*-like genes were identified on appropriate scaffolds in the draft genome of *A. chinensis* (Huang *et al.*, 2013) and subjected to BlastX analysis of the Arabidopsis peptide database. The identified Arabidopsis homologs (top blast hits) are listed according to the following colour code: green, genes in close proximity to *AGL14*/*AGL19*; red, close proximity to *SOC1*; blue, close proximity to *AGL42*/*AGL71*/*AGL72*.

Supplementary Table 1.

Oligonucleotide primers used for 5’RACE	
Name	Sequence
GR-SOC1b 3’	5’ CTAGCTAGGTCGTCGTTCAGGGGG 3’
GR-SOC1b 3’ Nested	5’ TCTGCGCTACTTGTAGGGTAATCGAC 3’
GR-SOC1e 3'	5’ TCACTTCTGCTGAGCTCGCTTG 3’
GR-SOC1e 3' Nested	5’ CTGGGCATGGCTCGTTCTGCC 3’
GR-SOC1f 3'	5’ TTATGCTAGGGTACGCTTGATTCTG 3’
GR-SOC1f 3' Nested	5’ TCTCACTACTTTCTGTTAATGGCACC 3’
GR-SOC1i 3'	5’ TCACTTCTGTGGAGCTCGATTG 3’
GR-SOC1i 3' Nested	5’ TTCTTTCCGCGGATGTAGGCAC 3’

Oligonucleotide primers used to amplify full-length kiwifruit *SOC1*-like genes	
Name	Sequence
attB1-AcSOC1a	5’ AAAAAGCAGGCTTCATGGTGAGAAGGAAGACAG 3’
attB2-AcSOC1a	5’ AGAAAGCTGGGTCTCATCGTTCGGGGGGTCG 3’
attB1-AcSOC1b	5’ AAAAAGCAGGCTTCATGGTGAGAGGGAAGACAGAG 3’
attB2-AcSOC1b	5’ AGAAAGCTGGGTCCTAGCTAGGTCGTCGTTC 3’
attB1-AcSOC1c	5’ AAAAAGCAGGCTTCATGGTGAGAGGGAAAACTG 3’
attB2-AcSOC1c	5’ AGAAAGCTGGGTCCTACCGGGTAGTTCGTGG 3’
attB1-SOC1d	5’ AAAAAGCAGGCTTCATGGTTAGAGGGAAAACTG 3’
attB2-SOC1d	5’ AGAAAGCTGGGTCCTACCTGGTAGTTCGTGGTTC 3’
attB2-SOC1e	5’ AGAAAGCTGGGTCTCACTTCTGCTGAGCTCG 3’
attB2-SOC1f	5’ AGAAAGCTGGGTCTTATGCTAGGGTACGCTTG 3’
attB2-SOC1g	5’ AGAAAGCTGGGTCTTATATATATAATGAGGTTAGGGTACG 3’
attB2-SOC1i	5’ AGAAAGCTGGGTCTCACTTCTGTGGAGCTCG 3’
attB1-SOC1e/f/g/i	5’ AAAAAGCAGGCTTCATGGTGAGAGGGAAGACT 3’
attB1-SOC1h	5’ AAAAAGCAGGCTTCATGGTGAGAGGAAAGGTTG 3’
attB2-SOC1h	5’ AGAAAGCTGGGTCTCATTTCAGCAAGGCCAAAG 3’

qRT-PCR primers for endogenous *SOC1* gene expression*	
Name	Sequence
qSOC1a-for	5’ GATGAACGAGCCGTTAATTAG 3’
qSOC1a-rev	5’ ATTTCATAGAAACACGGCTTCA 3’
qSOC1b-for	5’ GACCTAGCTAGCCGTTAACT 3’
qSOC1b-rev	5’ ACATTAGTACCCTTGGAAGCT 3’
qSOC1c-for	5’ AGCCTAGTCTTGTTCAATGAAT 3’
qSOC1c-rev	5’ GCTTTGGTACGTGTACTTTCA 3’
qSOC1d-for	5’ AGCATGTTATTGTCCAAAGTGT 3’
qSOC1d-rev	5’ TAATTTCCAGTGTATAAGCTCC 3’
TqSOC1e-for	5’ AAGCAAGGCCTAAACTCG 3’

qSOC1e-rev	5' GGGCCGATGACCAATTTGG 3'
qSOC1f-for	5' CGTACCCTAGCATAATCATCT 3'
qSOC1f-rev	5' TGCAATTCATCCATTACATAGC 3'
qSOC1g-for	5' AGCGTACCCTAACCTCATTAT 3'
qSOC1g-rev	5' GCTACACTGAATACATATAGTGT 3'
qSOC1h-for	5' GCTATTGGAGAAGGTATGGC 3'
qSOC1h-rev	5' AGCAGGTCATTTCAGCAAGG 3'
TqSOC1i-for	5' GGAAGTGCCTACATCCG 3'
qSOC1I-rev	5' GGGGTCCGATGAACAATTCA 3'

*, where possible primers were designed from the 3'UTR

qRT-PCR primers for *SOC1* transgene expression**

Name	Sequence
TqSOC1a-for	5' CAACATTAGATCAAGAAAGGA 3'
TqSOC1a-rev	5' GTAGCGTAGTTGATAAAAGCC 3'
TqSOC1b-for	5' ACATTAGGGCACGAAAGAG 3'
TqSOC1b-rev	5' GTAGGGTAATCGACAAAACCG 3'
TqSOC1c-for	5' GAAGTGTAAATGAAATGGGAAT 3'
TqSOC1c-rev	5' GTTCAATCTTCTCTGACATGTA 3'
TqSOC1d-for	5' GAAACGCAAAGAAACTAGGAAG 3'
TqSOC1d-rev	5' TTAATCTTCTCTAAAATCGTGG 3'
TqSOC1f-for	5' TGTTGTGGAGCGTGAAGCTT 3'
TqSOC1f-rev	5' TGTTGTCCAATCTTCTCACAC 3'
TqSOC1g-for	5' TGGAGCAAAGCCTGAAGCGC 3'
TqSOC1g-rev	5' CAATCTTCTCACGAATCTCACAC 3'
TqSOC1h-rev	5' TCATTTCAGCAAGGCCAAAG 3'
TqSOC1e/i/f-for	5' AGGAATTCGGTACCCCATCA 3'
TqSOC1e/i/f-rev	5' AAAGTCACTTGCCTGCTCGT 3'

** where qRT-PCR primers for endogenous *SOC1* gene expression not applicable

qRT-PCR primers for reference gene expression

Name	Sequence
qPP2A-for	5' GCAGCACATAATTCCACAGG 3'
qPP2A-rev	5' TTTCTGAGCCCATAACAGGAG 3'
qACTIN-for	5' CCAAGGCCAACAGAGAGAAG 3'
qACTIN-rev	5' GACGGAGGATAGCATGAGGA 3'
qUBC9-for	5' CCATTTCCAAGGTGTTGCTT 3'
qUBC9-rev	5' TACTTGTTCCGGTCCGTCTT 3'
qGAPDH-for	5' GCTATCAAGGAGGAATCAGAGG 3'
qGAPDH-rev	5' GCATCGAAGATGCTTGACCT 3'
qEF1alpha-for	5' GCACTGTCATTGATGCTCCT 3'
qEF1alpha-rev	5' CCAGCTTCAAAACCACCAGT 3'
RMW231 (AtACT2-for)	5' CTCTCCCGCTATGTATGTCGCCA 3'

RMW232 (AtACT2-rev)	5’ GTGAGACACACCATCACCAG 3’

Oligonucleotide primers used for genotyping	
Name	Sequence
AtSOC1-ATG-for	5' ATGGTGAGGGGCAAAACTCA 3'
AtSOC1-TDNA-for	5' TTGGGTTCACGTAGTGGGCCAT 3’
AtSOC1-intron-rev	5' CTGAAACATCTGATCAAAAGCTG 3'
RPH144	5’AGGAAGTTCATTTCATTTGGAGAGG 3’
HYG-rev	5’ CTGTTATGCGGCCATTGT 3’

Supplementary Table 2.

	SOC1	AcSOC1e	AcSOC1i	AcSOC1f	AcSOC1g	AGL14	AGL19	AcSOC1a	AcSOC1b	AcSOC1c	AcSOC1d	AGL42	AGL71	AGL72	AcSOC1h
SOC1		65.4	64.5	66.4	64.5	52.5	55.1	55.6	54.6	56	55.6	55.8	44.5	43.3	53.3
AcSOC1e	65.4		90.3	73	70.3	53.2	55.7	53.7	51.6	53	53	52.1	43.6	44.4	51.7
AcSOC1i	64.5	90.3		73	69.9	50	52.9	53.7	51.2	52.5	52.5	52.1	43.2	45.1	51.4
AcSOC1f	66.4	73	73		87.6	52.5	52.7	53.7	53	54.8	55.3	53.5	45	42.5	52.6
AcSOC1g	64.5	70.3	69.9	87.6		50.9	51.1	53.7	53.9	53.5	53.9	54	44.5	44.7	54.2
AGL14	52.5	53.2	50	52.5	50.9		76	54	53	53.5	54.1	51.9	42	43.5	47.9
AGL19	55.1	55.7	52.9	52.7	51.1	76		55.4	54.9	57.2	57.9	50.9	44.1	42.8	46.7
AcSOC1a	55.6	53.7	53.7	53.7	53.7	54	55.4		85.4	67	64.7	52.1	44.7	44.4	49.1
AcSOC1b	54.6	51.6	51.2	53	53.9	53	54.9	85.4		69.2	66	52.6	46.6	46.7	49.5
AcSOC1c	56	53	52.5	54.8	53.5	53.5	57.2	67	69.2		81.6	53.6	43.8	42.5	49.5
AcSOC1d	55.6	53	52.5	55.3	53.9	54.1	57.9	64.7	66	81.6		52.8	44.5	41.9	51.2
AGL42	55.8	52.1	52.1	53.5	54	50.9	51.9	52.1	52.6	53.6	52.8		51.6	49.5	56.2
AGL71	44.5	43.6	43.2	45	44.5	44.1	42	44.7	46.6	43.8	44.5	51.6		58.4	52.3
AGL72	43.3	44.4	45.1	42.5	44.7	42.8	43.5	44.4	46.7	42.5	41.9	49.5	58.4		50.5
AcSOC1h	53.3	51.7	51.4	52.6	54.2	47.9	46.7	49.1	49.5	49.5	51.2	56.2	52.3	50.5	

Supplementary Table 3.

Gene	AcSOC1a	AcSOC1b	AcSOC1c	AcSOC1d	AcSOC1e	AcSOC1f	AcSOC1g	AcSOC1i	AcSOC1h
Scaffold #	1	850	177	684	800	344	715	933	81
Gene models	Achn002551*	Achn361781*			Achn352151*	Ach196121 *			Achn354091*
					Achn352141*				
Top BlastX hit	AT4G11860 -	AT4G11820 -	AT4G11860 -	AT4G22920 -	AT4G31010 -	AT2G45670 -	AT2G45670 -	AT4G31010 -	AT5G51950 -
	AT4G22990 -	AT4G22920 +	AT1G62990 -	AT4G22900 -	AT2G42010 -	AT2G42010 -	AT2G42010 -	AT4G00230 -	AT5G51880 -
	AT1G63020 -	AT4G22910 +	AT4G11850 -	AT1G12560 -	AT2G45650 +	AT1G01910 -	AT1G01910 -	AT2G42010 -	AT5G51920 -
	AT4G23160 -	AT4G22880 +	AT4G38180 +	AT4G11970 -		AT1G01920 -	AT1G01920 -	AT2G45680 -	AT5G62230 -
	AT4G22910 +	AT1G12540 +	AT4G22900 +	AT1G04110 -		AT4G23010 -	AT4G23010 -	AT2G45690 -	AT5G51680 -
	AT4G22880 +	AT1G12530 +	AT1G12560 +	AT1G12520 -		AT2G29760 -	AT4G00770 -	AT1G01910 -	AT5G51670 -
	AT4G23160 +			AT4G11990 -		AT2G45720 -	AT1G01880 -	AT2G45650 +	AT5G51690 -
	AT1G12530 +			AT4G11860 +		AT1G01820	AT1G01860 -	AT2G45640 +	AT5G51660 -
				AT1G62990 +		AT3G26810 +	AT1G01840 -	AT2G46480 +	AT5G51600 -
				AT4G11850 +		AT5G13210 +	AT5G52040 -	AT4G25420 +	AT5G62170 +
				AT1G63000 +		AT1G12810 +	AT1G01820	AT2G45600 +	AT4G10260 +
				AT2G45680 +		AT2G33510 +	AT5G17680 -	AT2G45590 +	AT5G07200 +
				AT4G11820 +		AT5G13190 +	AT2G45750 -	AT2G45540 +	AT5G51780 +
						AT2G33490 +		AT2G45510 +	AT5G51800 +
									AT5G51790 +

Colour code: AGL14 (AT4G11880), AGL19 (AT4G22950), SOC1 (AT2G45660), AGL42 (AT5G62165), AGL71 (AT5G51870), AGL72 (AT5G51860)

*, incorrect gene model; -, upstream; +, downstream; underlined, homolog of AGL6, also in tandem with Arabidopsis SOC1 and peach PpSOC1
