# Supplementary material for: Functional and expression analyses of kiwifruit SOC1-like genes suggest that they may not have a role in the transition to flowering but may affect the duration of dormancy
Source: J Exp Bot. 2015 May 15;66(15):4699–710. doi: 10.1093/jxb/erv234 (PMC4507769; doi:10.1093/jxb/erv234)
Supplement: Supplementary Data [file supp_erv234_jexbot145029_file002.pdf]

SUPPLEMENTARY DATA

Figure S1. Alignment of the SOC1-like amino acid sequences. The amino acid residues in the alignment are coloured according to their similarity scores: white on black, 100% similar; white on grey, 80-100% similar; black on grey, 60-80% similar; grey on white, less than 60% similar. Origins of the predicted proteins, with the exception of Arabidopsis SOC1, AGL14, AGL19, AGL42, AGL71 and AGL72, are indicated by prefix as follows: *Ac*, *Actinidia chinensis*; *Vv*, *Vitis vinifera*; *Pt*, *Populus tremuloides*; *Ps*, *Pisum sativum*; *Ph*, *Petunia hybrida*; *Fv*, *Fragaria vesca*; *Pa*, *Prunus armeniaca*; *Md*, *Malus* x *domestica*.

Figure S2. Mean daily temperatures recorded during the collection of kiwifruit axillary bud samples.

Supplementary Figure S3. Protein interactions detected by yeast two-hybrid analysis. **(A)** Homodimerization of kiwifruit SOC1-like proteins. **(B)** Interaction of kiwifruit SOC1-like and Arabidopsis SOC1 proteins. **(C)** Heterodimerization of kiwifruit SOC1-like, SVP-like and Arabidopsis SOC1, SVP and AGL24. The final screen was performed on media lacking Trp, Leu and His and supplemented with indicated concentrations of 3-amino-1,2,4-triazole (3AT).

Supplementary Figure S4. RT-qPCR analysis of transgenic lines using primers specific to the *AcSOC1e* and *AcSOC1i* coding sequences and primers detecting the transgene expression. Primers sufficiently specific to *AcSOC1f* coding sequence could not be devised.

Figure S1

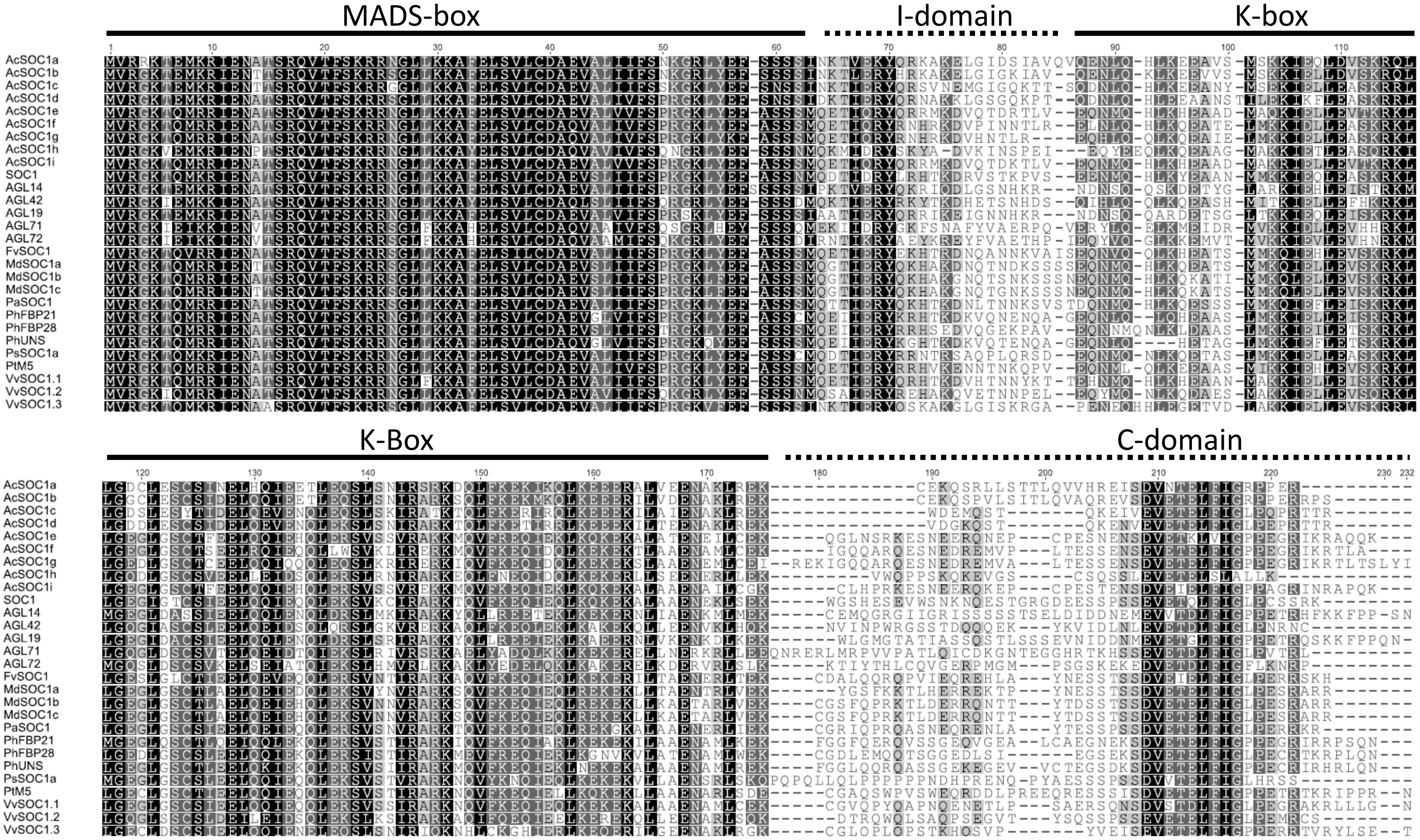
MADS-box
I-domain
K-box
K-Box
C-domain
AcSOC1a
AcSOC1b
AcSOC1c
AcSOC1d
AcSOC1e
AcSOC1f
AcSOC1g
AcSOC1h
AcSOC1i
SOC1
AGL14
AGL42
AGL19
AGL71
AGL72
FvSOC1
MdSOC1a
MdSOC1b
MdSOC1c
PaSOC1
PhFBP21
PhFBP28
PhUNS
PsSOC1a
PtM5
VvSOC1.1
VvSOC1.2
VvSOC1.3

Figure S2

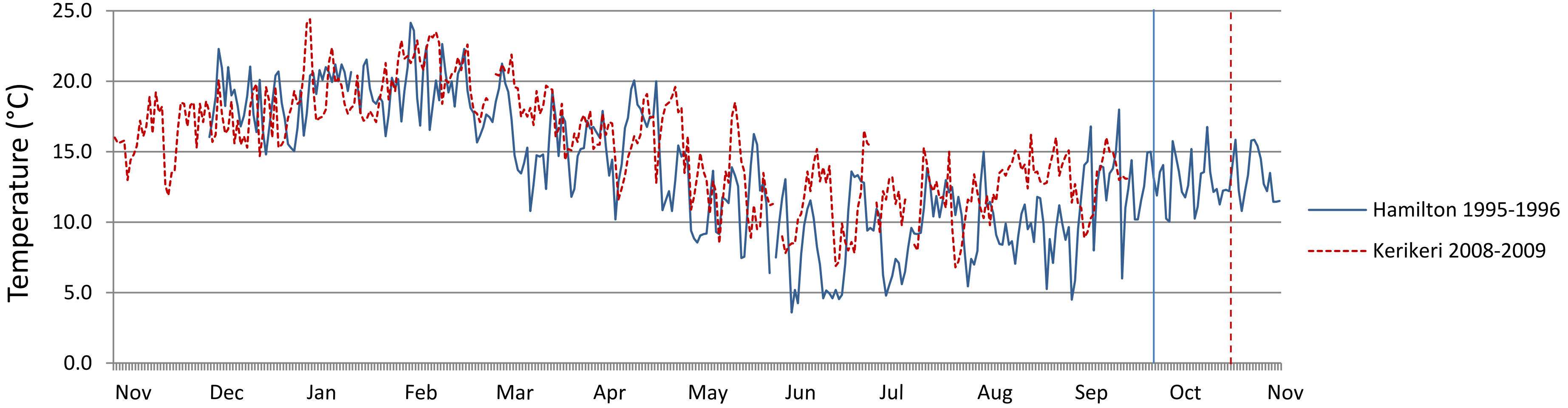
25.0
20.0
15.0
10.0
5.0
0.0
Temperature (°C)
Nov
Dec
Jan
Feb
Mar
Apr
May
Jun
Jul
Aug
Sep
Oct
Nov
Hamilton 1995-1996
Kerikeri 2008-2009

Figure S3

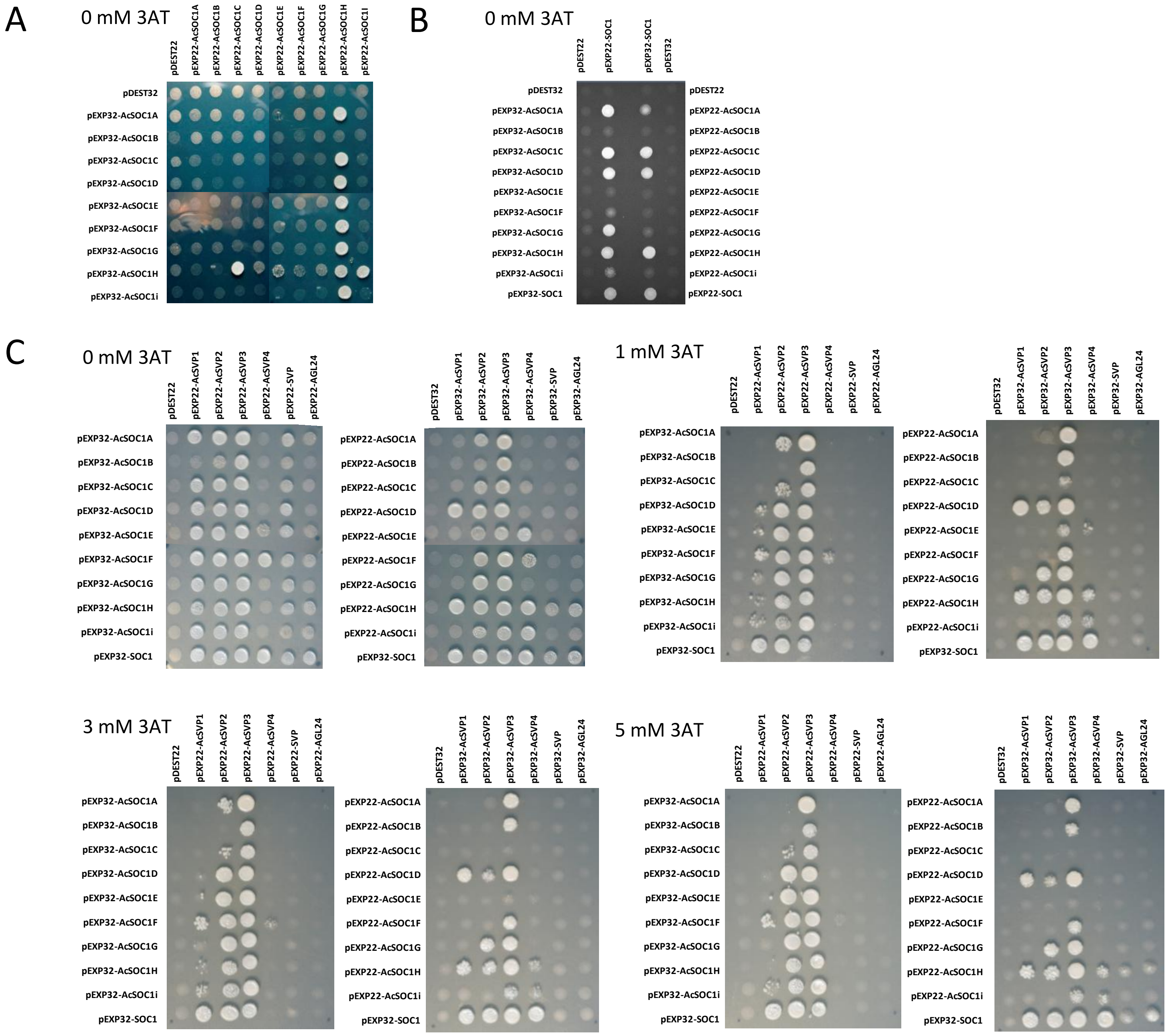
A
0 mM 3AT
pDEST22
pEXP22-AcSOC1A
pEXP22-AcSOC1B
pEXP22-AcSOC1C
pEXP22-AcSOC1D
pEXP22-AcSOC1E
pEXP22-AcSOC1F
pEXP22-AcSOC1G
pEXP22-AcSOC1H
pEXP22-AcSOC1I
pDEST32
pEXP32-AcSOC1A
pEXP32-AcSOC1B
pEXP32-AcSOC1C
pEXP32-AcSOC1D
pEXP32-AcSOC1E
pEXP32-AcSOC1F
pEXP32-AcSOC1G
pEXP32-AcSOC1H
pEXP32-AcSOC1i
B
0 mM 3AT
pDEST22
pEXP22-SOC1
pEXP32-SOC1
pDEST32
pDEST32
pEXP32-AcSOC1A
pEXP32-AcSOC1B
pEXP32-AcSOC1C
pEXP32-AcSOC1D
pEXP32-AcSOC1E
pEXP32-AcSOC1F
pEXP32-AcSOC1G
pEXP32-AcSOC1H
pEXP32-AcSOC1i
pEXP32-SOC1
pDEST22
pEXP22-AcSOC1A
pEXP22-AcSOC1B
pEXP22-AcSOC1C
pEXP22-AcSOC1D
pEXP22-AcSOC1E
pEXP22-AcSOC1F
pEXP22-AcSOC1G
pEXP22-AcSOC1H
pEXP22-AcSOC1i
pEXP22-SOC1
C
0 mM 3AT
1 mM 3AT
3 mM 3AT
5 mM 3AT
pDEST22
pEXP22-AcSVP1
pEXP22-AcSVP2
pEXP22-AcSVP3
pEXP22-AcSVP4
pEXP22-SVP
pEXP22-AGL24
pDEST32
pEXP32-AcSVP1
pEXP32-AcSVP2
pEXP32-AcSVP3
pEXP32-AcSVP4
pEXP32-SVP
pEXP32-AGL24
pEXP32-AcSOC1A
pEXP32-AcSOC1B
pEXP32-AcSOC1C
pEXP32-AcSOC1D
pEXP32-AcSOC1E
pEXP32-AcSOC1F
pEXP32-AcSOC1G
pEXP32-AcSOC1H
pEXP32-AcSOC1i
pEXP32-SOC1
pEXP22-AcSOC1A
pEXP22-AcSOC1B
pEXP22-AcSOC1C
pEXP22-AcSOC1D
pEXP22-AcSOC1E
pEXP22-AcSOC1F
pEXP22-AcSOC1G
pEXP22-AcSOC1H
pEXP22-AcSOC1i
pEXP32-SOC1

Figure S4

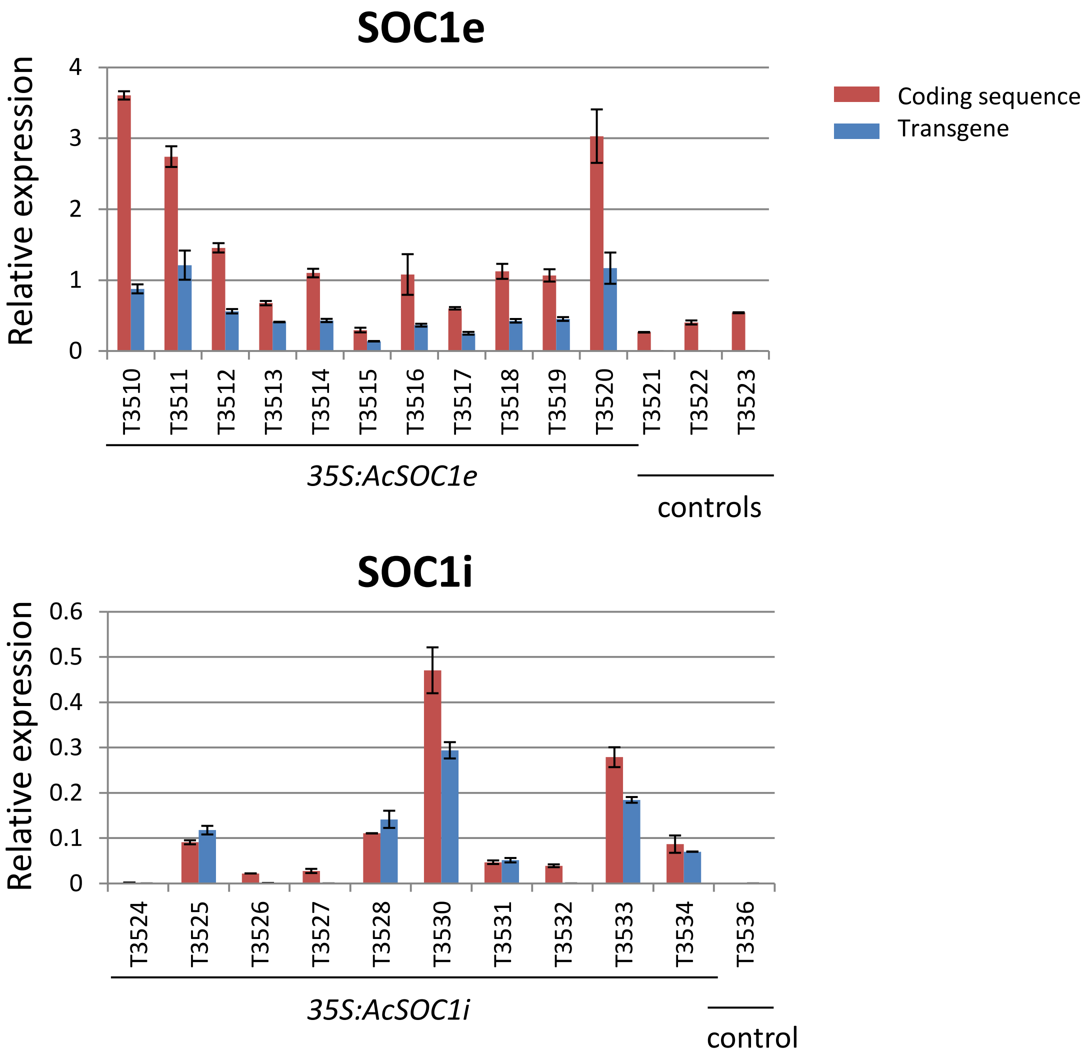
SOC1e
Relative expression
4
3
2
1
0
T3510
T3511
T3512
T3513
T3514
T3515
T3516
T3517
T3518
T3519
T3520
T3521
T3522
T3523
35S:AcSOC1e
controls
Coding sequence
Transgene
SOC1i
Relative expression
0.6
0.5
0.4
0.3
0.2
0.1
0
T3524
T3525
T3526
T3527
T3528
T3530
T3531
T3532
T3533
T3534
T3536
35S:AcSOC1i
control
